# The unintended health effects of US COVID-19 lockdowns: a systematic review

**DOI:** 10.1093/haschl/qxaf208

**Published:** 2025-10-30

**Authors:** Heather L Taylor, Pablo Cuadros, MaKenzie Gee, Nir Menachemi

**Affiliations:** Department of Health Policy, Richard M. Fairbanks School of Public Health, 1050 Wishard Blvd, Indianapolis, IN 46202, United States; Department of Health Policy, Richard M. Fairbanks School of Public Health, 1050 Wishard Blvd, Indianapolis, IN 46202, United States; Department of Health Policy, Richard M. Fairbanks School of Public Health, 1050 Wishard Blvd, Indianapolis, IN 46202, United States; Department of Health Policy, Richard M. Fairbanks School of Public Health, 1050 Wishard Blvd, Indianapolis, IN 46202, United States

**Keywords:** lockdown, school closure, COVID-19, health outcomes

## Abstract

**Introduction:**

US lockdowns and school closures implemented during the COVID-19 pandemic were intended to mitigate viral transmission and protect public health. However, the broader health effects of these interventions remain unclear.

**Methods:**

We conducted a systematic review of peer-reviewed studies that assessed the impact of US lockdowns and school closures on health-related outcomes excluding COVID-19 transmission and mortality.

**Results:**

A total of 132 studies met inclusion criteria, yielding 454 unique outcomes. Lockdowns and school closures were associated with detrimental health effects in the majority of outcomes analyzed, including over 90% of mental health, obesity-related, and health-related social need outcomes (child development/education, employment, access to food, and economic/financial stability). Analyses focused on vulnerable populations, such as racial and ethnic minorities, low-income groups, and individuals with disabilities, were significantly more likely to report detrimental outcomes than the general population.

**Conclusion:**

Given how lockdowns and school closures may affect population well-being, policymakers should carefully weigh both the benefits and harms of these interventions, including how they may affect vulnerable populations. We conclude with policy recommendations to mitigate ongoing harms and inform more evidence-based decision-making.

## Introduction

The sudden onset of the COVID-19 pandemic posed unprecedented challenges, forcing policymakers to make high-stakes decisions amid profound uncertainty. Limited information about the virus's transmission, risks, and severity left public health professionals and governments grappling with how best to respond. The United States, like other countries, prioritized reducing transmission by implementing “lockdowns,” an intervention that may include shelter-in-place/stay-at-home orders and the closure of schools and workplaces.^1,2^ Although intended to curb COVID-19 morbidity and mortality, emerging evidence suggests lockdowns may have adversely affected population health.^3-7^

In the United States, lockdowns were implemented despite the “very low quality” evidence regarding their effectiveness during previous pandemics/epidemics and the lack of information on potential unintended downstream consequences.^8-12^ Historically, public health authorities recommended lockdowns as a “last resort” in part because of their serious ethical, economic, health equity, and human rights concerns.^8,13-15^ While emerging evidence indicates that lockdowns reduced COVID-19 viral transmission^16,17^ and had little to no effect on COVID-19 mortality,^1,18,19^ far less attention has been given to the impact of lockdowns on broader health outcomes. Consequently, decision-makers in future pandemics are left with an incomplete picture, having evidence about the potential benefits of lockdowns (eg, reduced infections), but far less information on their potential drawbacks. Some studies analyzing global data suggest that lockdowns may have caused significant harm, including adverse effects on mental health, educational attainment, and socioeconomic conditions.^20-23^ However, the applicability of these global findings to the United States remains uncertain given the complex US political and social landscape^24^ and existing health and socioeconomic disparities experienced in the United States by vulnerable populations.^25^

To address this gap in the literature, we conducted a systematic review to examine the effects of US lockdowns including shelter-in-place/stay-at-home orders, workplace closures, and school closures on any other health outcome beyond their intended impact on COVID-19 transmission and mortality. We assess the breadth and strength of evidence regarding these consequences (either positive or negative), while considering factors such as study design, types of outcomes studied, and population characteristics. Further, we examine whether any health outcomes were disproportionately experienced by a wide range of vulnerable groups in US society. By identifying these broader impacts, our goal is to provide policymakers with a more comprehensive understanding of the full range of outcomes that may result following lockdown decisions. These insights contribute to the scientific understanding of the societal effects of such interventions and aim to equip future policymakers with more robust, evidence-based guidance for promoting equitable and effective pandemic responses.

## Methods

This systematic review was conducted and reported in accordance with the Preferred Reporting Items for Systematic Reviews and Meta-Analyses (PRISMA) guidelines.

### Search strategy

A comprehensive literature search was performed using the Medline and EMBASE bibliographic databases to identify applicable studies published between January 1, 2020 and December 31, 2024. The search included keywords such as “public health response”, “pandemic restriction”, “lockdown”, and “school closure”. The complete search strategy and list of keywords used can be found in Supplemental Material A.

### Inclusion criteria

Studies were eligible for inclusion if they met the following criteria: (1) published in English language peer-reviewed journals, (2) conducted on a US population or group, irrespective of age, (3) focused on the impact of COVID-19 lockdowns (defined as government-mandated stay-at-home/shelter-in-place orders and workplace closures) and/or school closures (defined as government or district-mandated temporary cessation of in-person K–12 instruction), and (4) assessed a health-related outcome other than COVID-19 transmission or mortality. We excluded studies that (1) lacked primary qualitative or quantitative data (eg, commentaries, letters to the editor, conference abstracts, systematic reviews, or meta-analyses) or (2) reported data from multiple countries without the ability to extract US-specific results.

### Study selection

Retrieved search results from both databases were de-duplicated. Three reviewers (H.T., P.C., and M.G.) independently screened the titles and abstracts of all articles. Full-text articles were then assessed for eligibility by at least 2 reviewers. Any disagreements regarding inclusion were resolved through discussion, with input from a fourth reviewer (N.M.) when necessary.

### Data extraction

A coding sheet was developed to extract key study characteristics and information from the abstracts of included studies. Prior to data extraction, the entire team met for training sessions designed to encourage questions, pilot-test the coding sheet, and reconcile any differences in interpretation and use of the coding sheet. A random sample of 15 papers was then independently coded by at least 2 reviewers. Inter-rater reliability across all extracted variables was high, with kappa statistics ranging from 0.72 to 1.0, indicating substantial to near-perfect agreement.^26^ The remaining studies were divided among 3 reviewers, who independently extracted data. Any questions or uncertainties that arose during independent data extraction were discussed and resolved in consultation with the full team.

For each included study abstract, we extracted information including all outcome variables that met our inclusion criteria as well as type of public health intervention studied (lockdown; school closures; or both), study design (simulation, qualitative, cross-sectional, pre-post, and quasi-experimental), and geographic characteristics of the study population (single state, multiple states, national). Because policy duration and type varied across states, we also recorded whether each study examined outcomes during the *initial national lockdown* (March–May 2020, relatively low variability) or an *extended period* (June 2020 and after, greater cross-state policy variability). For each unique outcome reported in included abstracts, we extracted data on whether the outcome was measured among a historically vulnerable or marginalized group (racial/ethnic minorities, non-English speaking/immigrants, sexual minority groups, low socioeconomic groups, uninsured/publicly insured, at-risk youth, those with disabilities, or the elderly). In cases where studies included results from multiple countries, the full manuscript was utilized to extract US-specific outcomes only.

For the main focus of our systematic review, we extracted the empirical results of every outcome analyzed and categorized the statistical findings based on the direction and significance of the associations. Outcomes were coded as “beneficial” if a study reported a statistically significant positive association for desirable outcomes (eg, increased sleep duration) or a statistically significant negative association for undesirable outcomes (eg, decreased substance use). Results were coded as “detrimental” if a study reported a statistically significant negative association for desirable outcomes (eg, reduced physical activity) or a statistically significant positive association for undesirable outcomes (eg, increased depressive symptoms). Results were classified as “null” if the study reported no statistically significant associations for the analyses of interest. In rare instances, and to be as conservative as possible, if the implications of a statistically significant effect could not be clearly determined (eg, decline in Google searches for “pregnancy test”),^27^ the result was coded as “unclear.” Finally, once all outcomes were extracted, 2 reviewers (HT & NM) met on an iterative basis to group outcomes into the following larger thematically-coherent categories: *access to food, access to health services, alcohol/drug/substance use, child development/education, disease-related, economic/financial stability, employment, family well-being, general health measures, general healthcare utilization, healthy behaviors, interpersonal violence/neglect/abuse, mental health, obesity, suicide or self-harm,* and *trauma/injury*.

### Analysis

We first examined the frequencies and percentages of study characteristics among all included studies. Next, we analyzed the distribution of statistical findings by outcome category (ie, detrimental, beneficial, null, or unclear). Using Chi-square tests, we assessed bivariate relationships between study characteristics and the likelihood of reporting detrimental outcomes. Analyses were further stratified by whether study outcomes focused on vulnerable populations and we evaluated the number of detrimental outcomes within each vulnerable group and outcome category. Finally, we qualitatively report findings from included studies with the strongest internal validity defined as those using quasi-experimental designs. We used Covidence software to aid in the management our work, and Stata 19.5 for all data analyses.

## Results

The search strategy retrieved 6329 unique articles (see Supplemental Material B). The full text of 496 studies was reviewed against inclusion/exclusion criteria after initial title and abstract screening. In total, 132 studies met the inclusion criteria (see Supplemental Material C) and were included in the current analysis. As shown in Table 1, most studies (*n* = 93, 70.4%) focused on lockdowns, while 27 (20.5%) examined school closures, and 12 (9.1%) examined both interventions. Fifty studies (37.9%) examined only the initial national lockdown (March–May 2020), 7 (5.3%) focused solely on the extended period (June 2020 and after), and 75 (56.8%) spanned both periods. The most common study design was pre/post (*n* = 84, 63.6%) or cross-sectional (*n* = 35, 26.5%). Less common were quasi-experimental (*n* = 6, 4.6%), qualitative (*n* = 4, 3.0%), and simulation (*n* = 3, 2.3%) study designs. Over half of the studies (*n* = 72, 54.5%) were conducted within a single state and 52 (39.4%) were national in scope. Nearly a third of the included studies (*n* = 42, 31.8%) examined impacts on vulnerable populations.

**Table 1.** Characteristics of included studies (*n* = 132).

	*N* (%)
Public health intervention	
Lockdown	93 (70.4%)
School-closure	27 (20.5%)
Both school closure and lockdown	12 (9.1%)
Lockdown Period	
Initial National Lockdown Period (March—May 2020)	50 (37.9%)
Extended period (June 2020 and after)	7 (5.3%)
Initial and extended period	75 (56.8%)
Study design	
Pre/post	84 (63.6%)
Cross-sectional	35 (26.5%)
Qualitative	6 (4.6%)
Quasi-experimental	4 (3.0%)
Simulation	3 (2.3%)
Age group of study subjects	
Adults (18 and older)	67 (50.8%)
Children/youth (17 or younger)	44 (33.3%)
Both children and adults	6 (4.5%)
Not specified	15 (11.4%)
Geography	
Single state	72 (54.5%)
National	52 (39.4%)
Multiple states	8 (6.1%)
Setting	
Urban	23 (17.4%)
Rural	3 (2.3%)
Both urban and rural	6 (4.5%)
Not specified	100 (75.8%)
Sentiment of authors in conclusion	
Lockdowns are negative/harmful	59 (44.7%)
Lockdowns are positive/beneficial	1 (0.8%)
Not specified/Unclear	72 (54.5%)
Number of outcomes analyzed	
Total across all studies	454
Mean (SD)	5.5 (3.4)
Median	5
Examined a vulnerable^a^ population	42 (31.8%)
Mean sample size (SD)	752 192 (3 365 057)
Sample Size Range	15-24,200,000

^a^Includes racial/ethnic minorities, non-English speaking individuals, sexual minority groups, low socioeconomic groups, uninsured/publicly insured, at-risk youth, those with disabilities, and the elderly.

Many authors examined more than one dependent variable in their study. As such, the 132 included studies contained a total of 454 outcome variables which were extracted and included in our analyses (see Table 2). Three fourths of outcomes (*n* = 339, 74.7%) were reported as detrimental. *Mental health* was the most frequently included category of outcomes (*n* = 68, 15.0% of all included outcomes), with 92.7% (*n* = 63) deemed detrimental. Other frequently studied outcome categories included *access to health services* (*n* = 60, 13.2% of all included outcomes), which had 75.0% of analyses (*n* = 45) reporting detrimental outcomes; and *alcohol, drug, or substance use* (*n* = 50, 11.0%), where 64.0% (*n* = 32) of statistical conclusions were detrimental. All, or nearly all, outcomes related to *obesity* (94.3%, *n* = 50 of 53), *child development/educational* (96.6%, *n* = 28 of 29), *employment* (100%, *n* = 11), *access to food* (100%, *n* = 9), and *economic/financial stability* (100%, *n* = 7) were statistically significantly detrimental. In contrast, categories like *trauma/injury* and *disease-related* outcomes showed a more mixed pattern of outcome effects with less than half of analyses reported as detrimental.

**Table 2.** Relationship between categories of outcomes and statistical conclusions of analyses (*n* = 454) reported in studies examining the effect of US COVID-19 lockdowns and/or school closures.

	Total outcomes analyzed	Detrimental	Beneficial	Null	Unclear
Type of category					
Mental health	68 (15.0%)	63 (92.7%)	3 (4.4%)	2 (2.9%)	—
Access to health services	60 (13.2%)	45 (75.0%)	5 (8.3%)	10 (16.7%)	—
Obesity	53 (11.7%)	50 (94.3%)	1 (1.9%)	2 (3.8%)	—
Alcohol/drug/substance use	50 (11.0%)	32 (64.0%)	10 (20.0%)	8 (16.0%)	—
Trauma/injury	46 (10.1%)	15 (32.6%)	15 (32.6%)	15 (32.6%)	1 (2.2%)
Healthy behaviors	40 (8.8%)	31 (77.5%)	6 (15.0%)	2 (5.0%)	1 (2.5%)
Child developmental/education	29 (6.4%)	28 (96.6%)	1 (3.4%)	—	—
Suicide or self-harm	22 (4.8%)	16 (72.7%)	4 (18.2%)	2 (9.1%)	—
Interpersonal violence/neglect/abuse	17 (3.7%)	10 (58.8%)	2 (11.8%)	3 (17.6%)	2 (11.8%)
General health measures	14 (3.1%)	9 (64.3%)	2 (14.3%)	3 (21.4%)	—
Disease-related	13 (2.9%)	6 (46.1%)	7 (53.9%)	—	—
Employment	11 (2.4%)	11 (100%)	—	—	—
Access to food	9 (2.0%)	9 (100%)	—	—	—
Family well-being	8 (1.8%)	2 (25.0%)	1 (12.5%)	2 (25.0%)	3 (37.5%)
General healthcare utilization	7 (1.6%)	5 (71.4%)	2 (28.6%)	—	—
Economic/financial stability	7 (1.6%)	7 (100%)	—	—	—
Total	454	339 (74.7%)	59 (13.0%)	49 (10.8%)	7 (1.5%)

Several study characteristics and outcomes were associated with a higher likelihood of reporting detrimental effects associated with lockdowns and/or school closures (see Table 3). Detrimental outcomes differed by study design, specifically cross-sectional (*n* = 95 of 112 outcomes; 84.8%) and qualitative (*n* = 20 of 22 outcomes; 90.9%) studies were more likely to report detrimental outcomes compared to pre/post (*n* = 197 of 285 outcomes; 69.1%) or quasi-experimental designs (*n* = 19 of 27 outcomes; 70.4%) (*P* = 0.002). Study designs with greater generalizability to the overall US population (ie, national or multi-state studies) were more likely to report detrimental effects than studies that were limited to locations in one state (84.1%, 85.0%, and 67.1%, respectively) (*P* < 0.001). Analyses of outcomes focused on vulnerable populations were significantly more likely to report detrimental effects than analyses focused on the general population (90.4% vs 70.0%, *P* < 0.001). In contrast, when comparing lockdowns to school closures, the rates of detrimental outcomes were similarly high, with no statistically significant difference observed (72.7% vs 80.7%, *P* = 0.285). There was also no statistically significant difference in the share of detrimental outcomes between studies limited to the initial national lockdown (75.4%) and those including the extended period (74.2%) (*P* = 0.771).

**Table 3.** Bivariate relationship between study characteristics and detrimental outcomes reported in studies examining the effects of US COVID-19 lockdowns and/or school closures.

Study characteristic	Non-detrimental outcome *N* (%)	Detrimental outcome *N* (%)	*P*-value
Public health intervention			
Lockdown (*n* = 330)	90 (27.3%)	240 (72.7%)	0.285
School closure (*n* = 83)	16 (19.3%)	67 (80.7%)	
Lockdown and school closure (*n* = 41)	9 (21.9%)	32 (78.1%)	
Lockdown period			
Initial national lockdown period (March—May 2020) (*n* = 167)	41 (24.6%)	126 (75.4%)	0.771
Included extended period (June 2020 and after) (*n* = 287)	74 (25.8%)	213 (74.2%)	
Study design			
Pre/post (*n* = 285)	88 (30.9%)	197 (69.1%)	0.002
Cross-sectional (*n* = 112)	17 (15.2%)	95 (84.8%)	
Qualitative (*n* = 22)	2 (9.1%)	20 (90.9%)	
Quasi-experimental (*n* = 27)	8 (29.6%)	19 (70.4%)	
Simulation (*n* = 8)	0 (0%)	8 (100%)	
Geography			
Single state (*n* = 252)	83 (32.9%)	169 (67.1%)	<0.001
National (*n* = 182)	29 (15.9%)	153 (84.1%)	
Multiple states (*n* = 20)	3 (15.0%)	17 (85.0%)	
Setting			
Urban (*n* = 90)	33 (36.7%)	57 (63.3%)	0.009
Rural (*n* = 8)	0 (0%)	8 (100%)	
Both urban & rural (*n* = 13)	5 (38.5%)	8 (61.5%)	
Not specified (*n* = 343)	77 (22.5%)	266 (77.5%)	
Vulnerable population^a^ studied			
Yes (*n* = 104)	10 (9.6%)	94 (90.4%)	<0.001
No (*n* = 350)	105 (30.0%)	245 (70.0%)	
Type of vulnerable group studied (*n* = 104)			
Racial/ethnic minorities (*n* = 29)	2 (6.9%)	27 (93.1%)	0.087
Low socioeconomic groups (*n* = 23)	1 (4.3%)	22 (95.7%)	
Those with disabilities (*n* = 11)	0 (0%)	11 (100%)	
At-risk youth (*n* = 11)	3 (27.3%)	8 (72.7%)	
Elderly (*n* = 9)	1 (11.1%)	8 (88.9%)	
Non-english speaking/immigrants (*n* = 7)	0 (0%)	7 (100%)	
Veterans (*n* = 5)	2 (40.0%)	3 (60.0%)	
Publicly insured/uninsured (*n* = 5)	0 (0%)	5 (100%)	
Sexual minority group (*n* = 4)	1 (25.0%)	3 (75.0%)	

^a^Includes outcomes that were reported as beneficial or null.

The most studied vulnerable population was racial and ethnic minorities (29 analyses), followed by low socioeconomic groups (23 analyses), at-risk youth (11 analyses) and individuals with disabilities (11 analyses) (see Material D). Among the 104 outcomes from included analyses focusing on vulnerable populations (see Table 4), the majority (*n* = 94, 90.4%) were reported detrimental effects. The most studied category was *access to health services* (*n* = 24, 23.1%), with 91.6% (*n* = 22 of 24) of outcomes deemed detrimental. Among analyses focused on any vulnerable population, one hundred percent of outcomes were found to be detrimental in categories such as *obesity* (*n* = 18 of 18), *economic/financial stability* (*n* = 6 of 6), *general health measures* (*n* = 5 of 5), *access to food* (*n* = 4 of 4), and *employment* (*n* = 2 of 2). Outcomes were also found to be predominantly detrimental in areas such as *mental health* (91.7%, *n* = 11 of 12), *healthy behaviors* (90.0%, *n* = 9 of 10) and *child development/education* (88.9%, *n* = 8 of 9).

**Table 4.** Relationship between categories of outcomes and statistical conclusions of analyses focused upon vulnerable populations^a^ (*n* = 104) from in studies examining the effects of US COVID-19 lockdowns and/or school closures.

	Total outcomes analyzed	Detrimental	Beneficial	Null	Unclear
Categories					
Access to health services	24 (23.1%)	22 (91.6%)	1 (4.2%)	1 (4.2%)	—
Obesity	18 (17.3%)	18 (100%)	—	—	—
Mental health	12 (11.5%)	11 (91.7%)	—	1 (8.3%)	—
Healthy behaviors	10 (9.6%)	9 (90.0%)	1 (10.0%)	—	—
Alcohol/drug/substance use	9 (8.6%)	6 (66.6%)	—	3 (33.3%)	—
Child developmental/education	9 (8.6%)	8 (88.9%)	1 (11.1%)	—	—
Economic/financial stability	6 (5.8%)	6 (100%)	—	—	—
General health measures	5 (4.8%)	5 (100%)	—	—	—
Access to food	4 (3.9%)	4 (100%)	—	—	—
Disease-related	2 (1.9%)	1 (50%)	1 (50.0%)	—	—
Employment	2 (1.9%)	2 (100%)	—	—	—
Suicide or self-harm	1 (1.0%)	1 (100%)	—	—	—
Family well-being	1 (1.0%)	—	1 (100%)	—	—
Trauma/injury	1 (1.0%)	1 (100%)	—	—	—
Interpersonal violence/neglect/abuse	0	—	—	—	—
General Healthcare Utilization	0	—	—	—	—
Total	104	94 (90.4%)	5 (4.8%)	5 (4.8%)	—

^a^Includes racial/ethnic minorities, non-English speaking individuals, sexual minority groups, low socioeconomic groups, uninsured/publicly insured, at-risk youth, those with disabilities, and the elderly.

Not all outcomes examined were consistently associated with detrimental effects. Findings related to the effect of lockdowns and school closures on trauma and certain diseases in the general population were more mixed. Approximately one-third of studies on trauma outcomes reported beneficial associations, such as reductions in motor vehicle-, pedestrian-, and motorcycle-related accidents and trauma admissions.^28,29^ Conversely, another third of trauma analyses reported detrimental associations, including increases in trauma-related admissions due to gun and knife violence.^30-32^ Among disease-related outcomes, lockdowns and school closures were associated with decreases in hospitalizations for respiratory conditions among children,^33^ but higher rates of late-stage lung cancer diagnoses among adults likely due to delayed or missed care.^34^

Of the 27 outcomes collected from included quasi experimental studies, 19 were statistically detrimental, 2 were beneficial, 2 were null, and 4 were unclear (See Material E). Brodeur (2021) found increased Google search activity for terms such as “boredom,” “loneliness,” “sadness,” and “worry,” suggesting mental health was “adversely affected.” ^35^ Cafferty (2024) reported a rise in the rate of positive suicide screenings among adolescents during the first pandemic year, with the trend reversing following school reopening.^36^ Weaver (2021) reported sharp increases in BMI z-scores among children during school closures, with an effect approximately tenfold greater than the mean yearly increase reported in pre-pandemic years (2017-2019).^37^ This increase was observed across gender, racial groups, and age, with the greatest weight gains experienced by children with normal pre-pandemic weight.^37^ Rapoport (2021) found a decrease in reported allegations of child maltreatment and child protective services investigations in New York City between March and May 2020.^38^ The authors hypothesized that this decline most likely reflected underreporting rather than a true reduction in incidence, as stay-at-home orders and school closures disrupted traditional surveillance and reporting mechanisms; however, this interpretation could not be directly confirmed from their data.^38^ Ferwana (2024) found consistent increases in mental health facility use during lockdowns and school closures, accounting for cross-state and within-county variation in policies and adjusting for local COVID-19 case counts.^39^ Diagnoses of panic disorder and severe stress also rose significantly during this period, further indicating detrimental mental health outcomes.^39^ Finally, Berger et al.^27^ analyzed shifts in Google search activity for terms related to family well-being, namely reproductive and family planning terms (“emergency contraceptive pill,” “pregnancy test,” “abortion,” and “condom”). The health implications of these behavioral shifts were classified as “unclear”.

## Discussion

Findings from this review indicate that both lockdowns and school closures were frequently associated with detrimental effects across multiple categories of health outcomes, including measures of *mental health, obesity,* and health-related social needs (ie, *child development/education, employment, food access,* and *economic/financial stability*). Our results are consistent with studies conducted outside the United States, which have similarly documented that these interventions were associated with increased rates of anxiety and depression,^22,40^ rising obesity,^41,42^ worsening food insecurity,^43-45^ declines in student achievement and learning,^23,46^ and disruptions to employment^47^ or economic stability.^48^ Notably, US lockdowns and school closures were associated with disproportionate harm among vulnerable populations, a finding also reported in global research.^5,20,49,50^

The disproportionate burden these measures place on vulnerable populations is likely driven by the effect of lockdowns and school closures on health-related social needs. Workplace closures disrupted employment and income, which in turn affected food and housing stability. Similarly, school closures limited children's access to both educational opportunities and essential nutrition through subsidized meal programs. While interventions aimed at protecting population health during crises may be necessary, those that undermine health-related social needs are particularly problematic given the well-established evidence demonstrating how these factors are stronger predictors of overall health than medical care or individual health behaviors.^51,52^ Because COVID-19 lockdowns and school closures disrupted health-related social needs, they may have exacerbated existing health inequities in the United States. Thus, when utilitarian aims, such as maximizing lives saved, seem to dominate infection control strategies during public health emergencies, it remains critical for policymakers to carefully consider the benefits, harms, and ethical implications of these policies even in times of uncertainty.^53,54^

Given our findings, public health decisions regarding lockdowns and school closures may have inadvertently violated the foundational principles of public health ethics that emphasize justice, equity, and protection of vulnerable populations.^53,54^ Key public health frameworks, including the Social Determinants of Health model, Maslow's Hierarchy of Needs, and the Socioecological Model,^55-57^ highlight the ethical and practical limitations of expecting individuals to comply with stay-at-home orders while their basic survival needs are unmet or become destabilized. Maslow's theory, for instance, underscores how social and economic disadvantage constrains an individual's capacity to prioritize health-related behaviors. As a result, when basic physiological and safety needs including food and economic stability are disrupted, vulnerable populations already living at or near the lowest tier of Maslow's hierarchy are more likely to focus on survival rather than health promotion. As such, policies that broadly undermine access to health-related social needs should be enacted only with extreme caution and swiftly reversed when emerging data shows that the true infection risk is far lower^58^ than early, often unreliable simulation models suggested.^59^

Beyond adverse effects on health-related social needs, lockdowns and school closures were associated with poor outcomes in mental health and obesity. Quasi-experimental studies reported an 18% increase in mental health facility use across all ages^39^ and a 19-fold increase in obesity risk among children previously classified at normal weight prepandemic.^37^ These data raise concerns about long-term population health and downstream burdens on health systems. Both poor mental health and obesity increase all-cause mortality and impose substantial financial burdens on communities.^60,61^ Furthermore, several studies also found that these negative outcomes were not evenly distributed, with Black and Hispanic populations disproportionately experiencing poorer mental health^62,63^ and worse obesity-related outcomes than White populations.^37,64-68^ These findings further suggest that lockdowns and school closures worsened existing health inequities, potentially leading to greater long-term harms.

The potential lasting effects of lockdowns and school closures such as rising childhood obesity, worsening mental health, and learning loss warrant policies focused on recovery efforts. Our findings point to the need for ongoing national and state-level policies that reverse these harms by strengthening mental health care, education, and social support systems, especially in communities hit hardest. In the future, policy decisions regarding pandemic restrictions should weigh health, economic, and educational outcomes openly and fairly, recognizing that protecting well-being involves more than preventing disease. Tools that compare different policy tradeoffs (such as quality-adjusted life years saved and lost) can help ensure that no group bears an unfair share of the burden. Future policies should also aim to be as least restrictive as possible, with clear equity safeguards (ie, ensuring that no community experiences substantially greater rates of food insecurity or youth mental health crises). When disparities widen, governments should pivot to less disruptive strategies or provide targeted supports. Importantly, a just and resilient approach must prioritize public engagement and transparency not just retrospectively, but proactively.^69^

Our study is the first to systematically quantify the broad health impacts of lockdowns and school closures in the United States. Nevertheless, several limitations should be acknowledged. First, similar to every individual paper included in this review, there is potential for confounding between the effects of lockdown policies and the effects of the pandemic itself. For example, worsening mental health during the pandemic may have reflected stay-at-home restrictions or fear of infection, bereavement, or stress among frontline workers. While this limitation applies broadly across studies in this area, some of the most rigorously designed analyses did attempt to address this concern. For instance, quasi-experimental work found that increases in mental health facility use were more strongly associated with the presence of lockdown policies than with the pandemic or illness itself,^39^ suggesting that policy restrictions exerted an independent effect on population well-being. Second, we recognize that our search strategy may have missed some health outcomes that could have been affected by lockdowns. Because our focus was limited to health-related outcomes, other important societal effects, such as changes in public trust or the viability of small businesses, were outside the scope of our study. Third, we extracted outcome data from study abstracts, not full manuscripts, which may have led to an undercounting of relevant outcomes. Similarly, we excluded studies that analyzed the general impact of the pandemic, but did not explicitly measure the effects of lockdowns or school closures as distinct exposures. This exclusion, while necessary for internal validity, may have omitted studies with relevant insights. Taken together, these limitations suggest our study likely underestimates the full effects of health consequences associated with lockdowns and/or school closures. Nonetheless, because not all outcomes (such as those related to trauma or disease) were consistently associated with detrimental effects, the overall distribution of findings helps mitigate concerns about publication bias within this review.

Beyond measuring broad health impacts, future research should also quantify the economic burden associated with lockdowns and school closures, and the total quality-adjusted life years saved and lost from imposing such measures. By using various metrics to evaluate the effect of these policies, decision-makers and public health professionals gain a better understanding of both the upsides and downsides of these interventions. In addition, our findings provide an opportunity for the broader field to reflect upon what level of infectious disease risk is ethically and justly appropriate to restrict access to education and the right to work, which are considered inalienable human rights directly linked to health and well-being.^70^

In conclusion, this systematic review generated evidence that can better inform public health professionals and decision-makers on broader health outcomes associated with lockdowns and school closures. Our findings suggest these interventions contributed to significant adverse health effects. Policymakers should carefully weigh the full spectrum of health consequences when considering future lockdown and school closure decisions, particularly ones that threaten to exacerbate existing health inequities among vulnerable groups.

## Supplementary Material

qxaf208_Supplementary_Data
