# Supplementary material for: The unintended health effects of US COVID-19 lockdowns: a systematic review
Source: Health Aff Sch. 2025 Oct 30;3(11):qxaf208. doi: 10.1093/haschl/qxaf208 (PMC12612676; doi:10.1093/haschl/qxaf208)
Supplement: qxaf208_Supplementary_Data [file qxaf208_supplementary_data.zip › Supplemental Material_U.S. COVID lockdowns_school closures.docx]

**Supplementary Material:** The unintended effects of U.S. COVID-19 Lockdowns: A systematic review

**Supplementary Material A – Search strategy**

Search strategy for Embase & Ovid (Medline)

| **1** | 'public health response'.mp. [mp=ti, bt, ab, ot, nm, hw, fx, kf, ox, px, rx, ui, an, sy, ux, mx, tn, dm, mf, dv, dq] |
| --- | --- |
| **2** | 'pandemic restriction'.mp. [mp=ti, bt, ab, ot, nm, hw, fx, kf, ox, px, rx, ui, an, sy, ux, mx, tn, dm, mf, dv, dq] |
| **3** | 'lockdown'.mp. [mp=ti, bt, ab, ot, nm, hw, fx, kf, ox, px, rx, ui, an, sy, ux, mx, tn, dm, mf, dv, dq] |
| **4** | 'school closure'.mp. [mp=ti, bt, ab, ot, nm, hw, fx, kf, ox, px, rx, ui, an, sy, ux, mx, tn, dm, mf, dv, dq] |
| **5** | exp Socioeconomic Factors |
| **6** | exp Food Insecurity |
| **7** | exp Hunger |
| **8** | "child maltreatment".mp. [mp=ti, bt, ab, ot, nm, hw, fx, kf, ox, px, rx, ui, an, sy, ux, mx, tn, dm, mf, dv, dq] |
| **9** | "alcohol dependence".mp. [mp=ti, bt, ab, ot, nm, hw, fx, kf, ox, px, rx, ui, an, sy, ux, mx, tn, dm, mf, dv, dq] |
| **10** | "alcohol use".mp. [mp=ti, bt, ab, ot, nm, hw, fx, kf, ox, px, rx, ui, an, sy, ux, mx, tn, dm, mf, dv, dq] |
| **11** | "Substance use".mp. [mp=ti, bt, ab, ot, nm, hw, fx, kf, ox, px, rx, ui, an, sy, ux, mx, tn, dm, mf, dv, dq] |
| **12** | "Suicide".mp. [mp=ti, bt, ab, ot, nm, hw, fx, kf, ox, px, rx, ui, an, sy, ux, mx, tn, dm, mf, dv, dq] |
| **13** | "Mental Illness".mp. [mp=ti, bt, ab, ot, nm, hw, fx, kf, ox, px, rx, ui, an, sy, ux, mx, tn, dm, mf, dv, dq] |
| **14** | "obesity".mp. [mp=ti, bt, ab, ot, nm, hw, fx, kf, ox, px, rx, ui, an, sy, ux, mx, tn, dm, mf, dv, dq] |
| **15** | "years of life lost".mp. [mp=ti, bt, ab, ot, nm, hw, fx, kf, ox, px, rx, ui, an, sy, ux, mx, tn, dm, mf, dv, dq] |
| **16** | "forgone health care".mp. [mp=ti, bt, ab, ot, nm, hw, fx, kf, ox, px, rx, ui, an, sy, ux, mx, tn, dm, mf, dv, dq] |
| **17** | "learning loss".mp. [mp=ti, bt, ab, ot, nm, hw, fx, kf, ox, px, rx, ui, an, sy, ux, mx, tn, dm, mf, dv, dq] |
| **18** | "school dropouts".mp. [mp=ti, bt, ab, ot, nm, hw, fx, kf, ox, px, rx, ui, an, sy, ux, mx, tn, dm, mf, dv, dq] |
| **19** | "domestic abuse".mp. [mp=ti, bt, ab, ot, nm, hw, fx, kf, ox, px, rx, ui, an, sy, ux, mx, tn, dm, mf, dv, dq] |
| **20** | "excess mortality".mp. [mp=ti, bt, ab, ot, nm, hw, fx, kf, ox, px, rx, ui, an, sy, ux, mx, tn, dm, mf, dv, dq] |
| **21** | 1 or 2 or 3 or 4 |
| **22** | 5 or 6 or 7 or 8 or 9 or 10 or 11 or 12 or 13 or 14 or 15 or 16 or 17 or 18 or 19 or 20 |
| **23** | 21 and 22 |
| **24** | Limit 23 to yr = “2000-2024” |
| **25** | Limit 24 to English language |

**Supplementary Material B – PRISMA Flow Diagram**

**
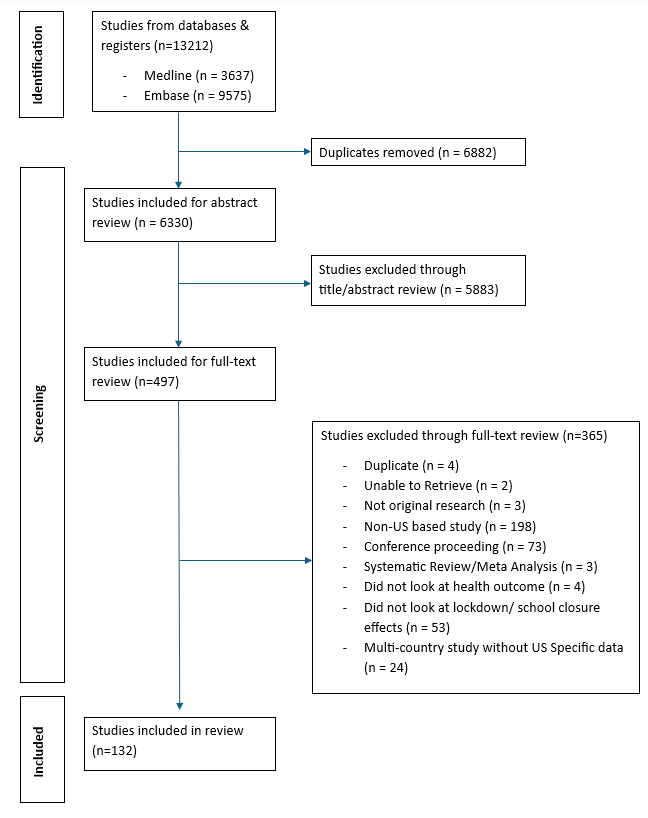
**

**Supplementary Material C – List of included studies**

1. Abidogun TM, Russell EC, Lindley LL, Griffin KW. Alcohol misuse among university students before and during COVID-19: The role of mental health. *J Am Coll Health*. 2025;73(4):1753-1760.

2. Adise S, West AE, Rezvan PH, et al. Socioeconomic disadvantage and youth mental health during the COVID-19 pandemic lockdown. *JAMA Netw Open*. 2024;7(7):e2420466.

3. Aghababian AH, Sadler JR, Jansen E, Thapaliya G, Smith KR, Carnell S. Binge watching during COVID-19: Associations with stress and body weight. *Nutrients*. 2021;13(10):3418.

4. Almandoz JP, Xie L, Schellinger JN, et al. Substance use, mental health and weight-related behaviours during the COVID-19 pandemic in people with obesity. *Clin Obes*. 2021;11(2):e12440.

5. An R. Projecting the impact of the coronavirus disease-2019 pandemic on childhood obesity in the United States: A microsimulation model. *J Sport Health Sci*. 2020;9(4):302-312.

6. Appelhans BM, French SA, Martin MA, Lui K, Janssen I. Attenuated efficacy of pediatric obesity treatment during the COVID-19 pandemic. *Obesity (Silver Spring)*. 2022;30(1):45-49.

7. Anand A, Weleff J, Thompson NR, Barnett BS. Prescribing of Z-drugs with and without opioid coprescribing to primary care patients in a large health care system from 2019-2020. *J Clin Psychiatry*. 2023;84(6). doi:10.4088/JCP.22m14753

8. Badach JM, Platoff R, Rattigan D, et al. Trauma incidence during the New Jersey COVID-19 stay-at-home order: A multicenter study. *J Surg Res*. 2023;284:264-268.

9. Baker BH, Day DB, Hazlehurst MF, Herkert NJ, Stapleton HM, Sathyanarayana S. Associations of environmental chemical exposures measured in personal silicone wristbands with sociodemographic factors, COVID-19 restrictions, and child respiratory health. *Environ Res*. 2024;262(Pt 1):119776.

10. Bao X, Qu H, Zhang R, Hogan TP. Modeling reading ability gain in kindergarten children during COVID-19 school closures. *Int J Environ Res Public Health*. 2020;17(17):6371.

11. Barbosa C, Cowell AJ, Dowd WN. Alcohol consumption in response to the COVID-19 pandemic in the United States. *J Addict Med*. 2021;15(4):341-344.

12. Bazzi AJ, Sallman ZF, Greenwell AM, Manolis AT, Khanafer R, Haidar-Elatrache S. Prolonged school closure and pediatric respiratory hospitalization: The silver lining of the COVID-19 pandemic. *Glob Pediatr Health*. 2024;11:2333794X231224999.

13. Berger LM, Ferrari G, Leturcq M, Panico L, Solaz A. COVID-19 lockdowns and demographically-relevant Google Trends: A cross-national analysis. *PLoS One*. 2021;16(3):e0248072.

14. Bernstein AN, Talwar R, Handorf E, et al. Impact of COVID-19 on initial management and evaluation of prostate cancer. *Urol Pract*. 2021;8(6):668-675.

15. Betts SS, Adise S, Hayati Rezvan P, et al. Socioeconomic adversity and weight gain during the COVID-19 pandemic. *JAMA Pediatr*. 2023;177(10):1102-1105.

16. Bliton JN, Paul J, Smith AD, et al. Increases in adolescent firearm injuries were associated with school closures during COVID-19. *Injury*. 2023;54(8):110824.

17. Blumenthal A. A bad time for kids in lockdown: The relationship between negative pandemic events, parenting stress, and maltreatment related parenting behaviors. *Child Abuse Negl*. 2023;138(106060):106060.

18. Borgatti AC, Schneider-Worthington CR, Stager LM, et al. The COVID-19 pandemic and weight management: Effective behaviors and pandemic-specific risk factors. *Obes Res Clin Pract*. 2021;15(5):518-521.

19. Brodeur A, Clark AE, Fleche S, Powdthavee N. COVID-19, lockdowns and well-being: Evidence from Google Trends. *J Public Econ*. 2021;193(104346):104346.

20. Bullinger LR, Raissian KM, Feely M, Schneider WJ. The neglected ones: Time at home during COVID-19 and child maltreatment. *Child Youth Serv Rev*. 2021;131(106287):106287.

21. Burnett D, Eapen V, Lin PI. Time trends of the public’s attention toward suicide during the COVID-19 pandemic: Retrospective, longitudinal time-series study. *JMIR Public Health Surveill*. 2020;6(4):e24694.

22. Burney JA, Roberts SC, DeHaan LL, et al. Epidemiological and clinical features of Kawasaki disease during the COVID-19 pandemic in the United States. *JAMA Netw Open*. 2022;5(6):e2217436.

23. Cafferty R, Haasz M, Leonard J, Ambroggio L. Impact of COVID-19 and public health measures on positive suicide screens among emergency department children. *Pediatr Emerg Care*. 2024;40(9):e227-e232.

24. Cantor J, Whaley CM, Ward J, Jena AB. COVID-19 school closures were associated with A decline in employment for female nurses with young children: Article examines impact of COVID-19 school closures on female nurses with young children. *Health Aff (Millwood)*. 2024;43(9):1329-1337.

25. Casassa C, Moss R, Goldenholz DM. Epilepsy during the COVID-19 pandemic lockdown: a US population survey. *Epileptic Disord*. 2021;23(2):257-267.

26. Chakrabarti S, Hamlet LC, Kaminsky J, Subramanian SV. Association of human mobility restrictions and race/ethnicity-based, sex-based, and income-based factors with inequities in well-being during the COVID-19 pandemic in the United States. *JAMA Netw Open*. 2021;4(4):e217373.

27. Chatzoglou E, Fishstrom S, Payne SB, Andress TT, Vaughn S. The footprint of the COVID-19 pandemic in reading performance of students in the U.S. with and without disabilities. *Res Dev Disabil*. 2023;140(104585):104585.

28. Chiba H, Lewis M, Benjamin ER, et al. “Safer at home”: The effect of the COVID-19 lockdown on epidemiology, resource utilization, and outcomes at a large urban trauma center. *J Trauma Acute Care Surg*. 2021;90(4):708-713.

29. Cholankeril R, Xiang E, Badr H. Gender differences in coping and psychological adaptation during the COVID-19 pandemic. *Int J Environ Res Public Health*. 2023;20(2):993.

30. Christakis DA, Van Cleve W, Zimmerman FJ. Estimation of US children’s educational attainment and years of life lost associated with primary school closures during the Coronavirus disease 2019 pandemic. *JAMA Netw Open*. 2020;3(11):e2028786.

31. Christopoulos K. Associations between lockdown intensity and suicide mortality in US states. *SSM Popul Health*. 2023;24(101544):101544.

32. Conroy DA, Hadler NL, Cho E, et al. The effects of COVID-19 stay-at-home order on sleep, health, and working patterns: a survey study of US health care workers. *J Clin Sleep Med*. 2021;17(2):185-191.

33. Das A, Singh P, Bruckner TA. State lockdown policies, mental health symptoms, and using substances. *Addict Behav*. 2022;124(107084):107084.

34. Dellazoppa A, Porada K, Zaspel JA, Bourgeois S, Vepraskas SH. Impact of COVID-19 on acute care hospitalizations for suicidality. *Hosp Pediatr*. 2024;14(5):376-384.

35. Dietz WH. The COVID-19 lockdown increased obesity disparities; will the increases in type 2 diabetes continue? *Obesity (Silver Spring)*. 2023;31(3):699-702.

36. Duncan A, Herrera CN, Okobi M, Nandi S, Oblath R. Locked down or locked out? Trends in psychiatric emergency services utilization during the COVID-19 pandemic. *J Health Serv Res Policy*. 2023;28(2):80-88.

37. Dvir Y, Ryan C, Lee J. School closures and ED visits for suicidality in youths before and during the COVID-19 pandemic. *JAMA Netw Open*. 2023;6(11):e2343001.

38. Eden CM, Zhu R, Khedr S, Khariton K. Effect of the Coronavirus disease 2019 pandemic on suicide-related trauma burden at a level 1 trauma center. *J Emerg Trauma Shock*. 2022;15(2):88-92.

39. Ferns SJ, Gautam S, Hudak ML. COVID-19 and gender disparities in Pediatric Cardiologists with dependent care responsibilities. *Am J Cardiol*. 2021;147:137-142.

40. Ferwana I, Varshney LR. The impact of COVID-19 lockdowns on mental health patient populations in the United States. *Sci Rep*. 2024;14(1):5689.

41. Fleming M, Lee D, Oranu C, et al. Positive changes in safety perception among Blacks with HIV and comorbidities: Assessment of social determinants of health during COVID-19. *J Racial Ethn Health Disparities*. 2024;11(3):1578-1586.

42. Gallardo M, Zepeda A, Biely C, et al. School-based health center utilization during COVID-19 pandemic-related school closures. *J Sch Health*. 2022;92(11):1045-1050.

43. Galvan B, Holder KG, Boeger B, et al. Impact of COVID-19 pandemic at a level 1 trauma center. *Surg Pract Sci*. 2023;14(100189):100189.

44. George MP, Germack HD, Goyal A, Ward C, Studer S, Panjabi S. Impact of the COVID-19 pandemic on care disruptions, outcomes, and costs in patients receiving pulmonary arterial hypertension-specific therapy in the United States of America: An observational study. *Pulm Circ*. 2023;13(3):e12283.

45. Gillory L, Cairo S, Megison S, Vinson L, Chung DH, Ryan ML. Effect of quarantine and reopening measures on pediatric trauma admissions during the 2019 SARS-CoV2 virus pandemic. *J Am Coll Surg*. 2022;234(4):685-690.

46. Gimbrone C, Rutherford C, Kandula S, et al. Associations between COVID-19 mobility restrictions and economic, mental health, and suicide-related concerns in the US using cellular phone GPS and Google search volume data. *PLoS One*. 2021;16(12):e0260931.

47. Han M, Preciado P, Thwin O, et al. Effect of statewide lockdown in response to COVID-19 pandemic on physical activity levels of hemodialysis patients. *Blood Purif*. 2021;50(4-5):602-609.

48. Hasratian AM, Nordberg HO, Meuret AE, Ritz T. Fear and coping in students during the early stages of the COVID-19 pandemic: A combined cross-sectional and longitudinal study. *Int J Environ Res Public Health*. 2021;18(12):6551.

49. Hawrilenko M, Kroshus E, Tandon P, Christakis D. The association between school closures and child mental health during COVID-19. *JAMA Netw Open*. 2021;4(9):e2124092.

50. He Z, Heess JM, Young T, Lei Z. Lessons for future pandemics: Temporal evolution and rural-urban variations in the impacts of the COVID-19 on opioid use treatment. *PLoS One*. 2024;19(9):e0310386.

51. Hecht AA, Dunn CG, Kinsey EW, et al. Estimates of the nutritional impact of non-participation in the National School Lunch Program during COVID-19 school closures. *Nutrients*. 2022;14(7):1387.

52. Herzig SE, Albers L, Soto D, et al. Pandemic-related life changes and adolescent initiation of cannabis and tobacco/nicotine use. *Addict Behav*. 2023;144(107724):107724.

53. Horbinski C, Zumpf KB, McCortney K, Eoannou D. Longitudinal observational study of boxing therapy in Parkinson’s disease, including adverse impacts of the COVID-19 lockdown. *BMC Neurol*. 2021;21(1):326.

54. Hsieh T, Gundlach BS, Ashrafzadeh S, Sarraf D, Tsui I. Effects of COVID-19 on Intravitreal Injection Clinic After Lockdown. *Clinical ophthalmology (Auckland, NZ)*. 2022;16. doi:10.2147/opth.s358239

55. Hu A, Harvey A, Rogers AM, Rigby A, Butt M. Associations of COVID-19 lockdowns on eating behaviors and body mass index in patients with a history of bariatric surgery: A cross-sectional analysis. *Obes Surg*. 2023;33(4):1099-1107.

56. Huang EY, Grunvald E, Blitzer RR, et al. Despite regression of healthy lifestyle habits, weight loss after bariatric surgery is not affected during the COVID-19 pandemic. *Surg Endosc*. 2023;37(1):607-612.

57. Hunter RF, Garcia L, de Sa TH, et al. Effect of COVID-19 response policies on walking behavior in US cities. *Nat Commun*. 2021;12(1):3652.

58. Jagtiani P, Young T, Ahmed W, Devarajan A, Hickman ZL, Jones S. Prevalence of acute alcohol use in traumatic brain injury patients during the COVID-19 pandemic: A retrospective analysis from Queens, New York. *Cureus*. 2024;16(4):e58928.

59. Jiang B, Liu Z, Shen R, Huang L, Tong Y, Xia Y. Have COVID-19-related economic shocks affected the health levels of individuals in the United States and the United Kingdom? *Front Public Health*. 2020;8:611325.

60. Kämpfen F, Kohler IV, Ciancio A, Bruine de Bruin W, Maurer J, Kohler HP. Predictors of mental health during the Covid-19 pandemic in the US: Role of economic concerns, health worries and social distancing. *PLoS One*. 2020;15(11):e0241895.

61. Khan MMM, Munir MM, Woldesenbet S, et al. Association of COVID-19 pandemic with colorectal cancer screening: Impact of race/ethnicity and social vulnerability. *Ann Surg Oncol*. 2024;31(5):3222-3232.

62. Kim J, Rao N, Collins A, et al. Retrospective study of psychiatric hospitalizations in a West Texas mental health treatment facility during the COVID-19 pandemic. *South Med J*. 2023;116(2):170-175.

63. Kim Y, Krause TM, Lane SD. Trends and seasonality of emergency department visits and hospitalizations for suicidality among children and adolescents in the US from 2016 to 2021. *JAMA Netw Open*. 2023;6(7):e2324183.

64. Kim PC, Cochran C, Bai B, et al. Increasing emotional distress and use of health services among hospitality industry workers during and after the COVID-19 lockdown. *Ethn Dis*. 2024;34(2):75-83.

65. Knauft K, Zilioli S, Tarraf W, Rorai V, Perry TE, Lichtenberg PA. Social connectedness in older Urban African-American adults during the COVID-19 pandemic: the roles of education and partnership. *Aging Ment Health*. 2024;28(6):874-881.

66. Koebnick C, Sidell MA, Li X, et al. Disparities in weight changes during the COVID-19 pandemic-related lockdown in youths. *Obesity (Silver Spring)*. 2023;31(3):789-801.

67. Kothadia S, Chung W, Min M, Saeed F, Scharfen J, Habr F. Increased prevalence of alcohol-related gastrointestinal and liver diseases during the COVID-19 pandemic. *R I Med J (2013)*. 2022;105(10):57-62.

68. Krzyzaniak A, Carroll AN, Rooney AS, Calvo RY, Bansal V, Sise MJ. Firearm assaults in communities: The impact of COVID-19 lockdown. *Am Surg*. 2023;89(10):4200-4207.

69. Lee SJ, Ward KP, Lee JY, Rodriguez CM. Parental social isolation and child maltreatment risk during the COVID-19 pandemic. *J Fam Violence*. 2022;37(5):813-824.

70. Lee SJ, Ward KP, Rodriguez CM. Longitudinal analysis of short-term changes in relationship conflict during COVID-19: A risk and resilience perspective. *J Interpers Violence*. 2022;37(15-16):NP14239-NP14261.

71. Leichtle SW, Rodas EB, Procter L, Bennett J, Schrader R, Aboutanos MB. The influence of a statewide “Stay-at-Home” order on trauma volume and patterns at a level 1 trauma center in the united states. *Injury*. 2020;51(11):2437-2441.

72. Levitin HW, Jones BG, Lockhart MM, et al. Where have all the FLOWERS gone? A multicenter investigation of frequent users of Midwest emergency department services during the COVID-19 stay-at-home orders. *West J Emerg Med*. 2022;23(5):724-733.

73. Li Y, Huang Y, Li R, Zhang K. Historical redlining and park use during the COVID-19 pandemic: Evidence from big mobility data. *J Expo Sci Environ Epidemiol*. 2024;34(3):399-406.

74. Liu W, Li J, Dalton CM. Disruptions to in-person medical visits across the United States during the COVID-19 pandemic: evolving disparities by medical specialty and socio-economic status. *Public Health*. 2023;221:116-123.

75. Liu J, Chai L, Zhu H, Han Z. COVID-19 impacts and adolescent suicide: The mediating roles of child abuse and mental health conditions. *Child Abuse Negl*. 2023;138(106076):106076.

76. Liu Y, Arora T, Zhang J, Sodhi SK, Xie F, Curtis JR. The interruption of romosozumab treatment during COVID lockdown among US post-menopausal women enrolled in Medicare. *Bone*. 2024;178(116954):116954.

77. Lopez LD, Castillo A, Frechette E, et al. High-quality early care and education for low-income families: Toddlers’ cognitive and emotional functioning during the COVID-19 pandemic. *Infancy*. 2024;29(6):983-1001.

78. Madhok DY, Nardone A, Caceres EU, Wong AHK, Zhang L, Rodriguez RM. The impact of the COVID-19 shelter-in-place order on traumatic brain injuries in San Francisco, California. *J Emerg Med*. 2023;65(6):e479-e486.

79. Maxwell SL, McCulloch CE, Fernandez A, Beck AL. Changes in BMI prior to and during the COVID-19 pandemic among children: a retrospective cohort study in San Francisco, CA. *BMC Public Health*. 2024;24(1):2962.

80. McLaren RA Jr, Trejo FE, Blitz MJ, et al. COVID-related “lockdowns” and birth rates in New York. *Am J Obstet Gynecol MFM*. 2021;3(6):100476.

81. Messiah SE, Uppuluri M, Xie L, et al. Substance use, mental health, and weight-related behaviors during the COVID-19 pandemic among metabolic and bariatric surgery patients. *Obes Surg*. 2021;31(8):3738-3748.

82. Mitchell TO, Li L. State-level data on suicide mortality during COVID-19 quarantine: Early evidence of a disproportionate impact on racial minorities. *Psychiatry Res*. 2021;295(113629):113629.

83. Mogasala S, Helzer C, Welch RD, Secord E, McGrath E. Impact of the COVID-19 pandemic on BMI in youth living with HIV. *SAGE Open Med*. 2024;12:20503121241267080.

84. Moody RL, Chen YT, Schneider JA, et al. Polysubstance use in a community sample of Black cisgender sexual minority men and transgender women in Chicago during initial COVID-19 pandemic peak. *Subst Abuse Treat Prev Policy*. 2022;17(1):4.

85. Mulugeta W, Hoque L. Impact of the COVID-19 lockdown on weight status and associated factors for obesity among children in Massachusetts. *Obes Med*. 2021;22(100325):100325.

86. Mulugeta W, Desalegn H, Solomon S. Impact of the COVID-19 pandemic lockdown on weight status and factors associated with weight gain among adults in Massachusetts. *Clin Obes*. 2021;11(4):e12453.

87. Mynard N, Saxena A, Mavracick A, et al. Lung cancer stage shift as a result of COVID-19 lockdowns in New York City, a brief report. *Clin Lung Cancer*. 2022;23(3):e238-e242.

88. Naran V, Namous N, Eddy VJ, Le Guen CL, Sarwer DB, Soans RS. The effects of the COVID-19 pandemic on patients with obesity undergoing bariatric care. *Surg Obes Relat Dis*. 2021;17(10):1714-1720.

89. Noel JK, Rosenthal SR, Skierkowski-Foster D, Borden SK. Effect of COVID-19 lockdown on substance use among middle school and high school students: A natural experiment. *Public Health Rep*. 2023;138(2):349-356.

90. Nourazari S, Davis SR, Granovsky R, et al. Decreased hospital admissions through emergency departments during the COVID-19 pandemic. *Am J Emerg Med*. 2021;42:203-210.

91. Oblath R, Oh A, Herrera CN, Duncan A, Zhen-Duan J. Psychiatric emergencies among urban youth during COVID-19: Volume and acuity in a multi-channel program for the publicly insured. *J Psychiatr Res*. 2023;160:71-77.

92. Parker J, Kaur S, Medalla JM, Imbert-Sanchez A, Bautista J. Dietary trends among young adults during the COVID-19 lockdown: socioeconomic and gender disparities. *BMC Nutr*. 2023;9(1):107.

93. Patel NS, Waibel BH, Berning BJ, et al. Kids gone wild - Alcohol use and patient characteristics in pediatric trauma during the coronavirus disease 2019 pandemic. *Pediatr Investig*. 2023;7(4):225-232.

94. Payán DD, Perez-Lua F, Goldman-Mellor S, Young MEDT. Rural household food insecurity among Latino immigrants during the COVID-19 pandemic. *Nutrients*. 2022;14(13):2772.

95. Perry KJ, Perhamus GR, Lent MC, Murray-Close D, Ostrov JM. The COVID-19 pandemic and measurement of preschoolers’ executive functions. *Psychol Assess*. 2023;35(11):986-999.

96. Perry L, Scheerens C, Greene M, et al. Unmet health-related needs of community-dwelling older adults during COVID-19 lockdown in a diverse urban cohort. *J Am Geriatr Soc*. 2023;71(1):178-187.

97. Piquero AR, Riddell JR, Bishopp SA, Narvey C, Reid JA, Piquero NL. Staying home, staying safe? A short-term analysis of COVID-19 on Dallas domestic violence. *Am J Crim Justice*. 2020;45(4):601-635.

98. Plank K, Hewawitharana S, Talmage E, Rauzon S, Woodward-Lopez G. School meal access and changes in meal participation during COVID-19 school closures: A stratified random sample of CalFresh Healthy Living Eligible school districts in California. *Prev Med Rep*. 2022;28(101794):101794.

99. Press SG. Maxillofacial trauma and COVID-19: A review of the first 6 months of the pandemic. *Craniomaxillofac Trauma Reconstr*. 2022;15(1):34-38.

100. Pugh T, Harris J, Jarnagin K, Thiese MS, Hegmann KT. Impacts of the statewide COVID-19 lockdown interventions on excess mortality, unemployment, and employment growth. *J Occup Environ Med*. 2022;64(9):726-730.

101. Rapoport E, Reisert H, Schoeman E, Adesman A. Reporting of child maltreatment during the SARS-CoV-2 pandemic in New York City from March to May 2020. *Child Abuse Negl*. 2021;116(Pt 2):104719.

102. Rezai R, Hayati Rezvan P, Comulada WS, et al. Alcohol misuse among youth living with and at high risk for acquiring HIV during the COVID-19 stay-at-home orders: A study in Los Angeles and New Orleans. *Alcohol Alcohol*. 2023;58(3):238-246.

103. Romano E, Sánchez M. A characterization of pre- to post-immigration alcohol use among recent Latino immigrants to the United States before and during the COVID-19 pandemic. *Alcohol Clin Exp Res (Hoboken)*. 2023;47(2):296-307.

104. Roulette CJ, Kopels M. Perception of uncontrollable mortality risk is associated with food insecurity and reduced economic effort among resource-insecure college students during COVID-19. *Am J Hum Biol*. 2024;36(9):e24081.

105. Ruiz-Medina PE, Ramos-Meléndez EO, Cruz-De La Rosa KX, et al. The effect of the lockdown executive order during the COVID-19 pandemic in recent trauma admissions in Puerto Rico. *Inj Epidemiol*. 2021;8(1):22.

106. Sadegh-Zadeh SA, Bahrami M, Najafi A, Asgari-Ahi M, Campion R, Hajiyavand AM. Evaluation of COVID-19 pandemic on components of social and mental health using machine learning, analysing United States data in 2020. *Front Psychiatry*. 2022;13:933439.

107. Sanaka H, Garg R, Patel V, McMichael J, Macaron C. Temporal trends and disparities in gastroenterology care use before, during, and after COVID-19 lockdown. *J Investig Med*. 2022;70(8):1704-1712.

108. Sano E, Benton E, Kenny J, Olsen E, Heravian A, Truong J. Telemedicine use by older adults in a COVID-19 epicenter. *J Emerg Med*. 2022;63(3):325-331.

109. Scarfe ML, Haik AK, Rahman L, et al. Impact of COVID-19 on alcohol use disorder recovery: A qualitative study. *Exp Clin Psychopharmacol*. 2023;31(1):148-162.

110. Schwartz-Mette RA, Duell N, Lawrence HR, Balkind EG. COVID-19 distress impacts adolescents’ depressive symptoms, NSSI, and suicide risk in the rural, northeast US. *J Clin Child Adolesc Psychol*. 2023;52(5):702-715.

111. Sheehan C, Li L, Petrov ME. How did trends in sleep duration in 2020 compare to previous years and how did they vary by sex, race/ethnicity, and educational attainment? *Sleep Med*. 2023;101:570-577.

112. Sielaty R, Boutzoukas AE, Zimmerman KO, et al. Trends in pediatric emergency and inpatient healthcare use for mental and behavioral health among north carolinians during the early COVID-19 pandemic. *J Pediatric Infect Dis Soc*. 2023;12(Supplement_2):S20-S27.

113. Silverman ME, Sami TJ, Kangwa TS, Burgos L, Stern TA. Socioeconomic disparity in birth rates during the COVID-19 pandemic in New York city. *J Womens Health (Larchmt)*. 2022;31(8):1113-1119.

114. Singh K, Armstrong SC, Wagner BE, et al. Physical activity and sleep changes among children during the COVID-19 pandemic. *NPJ Digit Med*. 2024;7(1):70.

115. Srinagesh A, Forthal S, Madden SP, Stein LAR, Muench F. Impacts of COVID-19 on alcohol use among help-seeking adults. *Adv Drug Alcohol Res*. 2023;3:11159.

116. Stephenson R, Chavanduka TMD, Rosso MT, et al. Sex in the Time of COVID-19: Results of an online survey of gay, bisexual and other men who have sex with men’s experience of sex and HIV prevention during the US COVID-19 epidemic. *AIDS Behav*. 2021;25(1):40-48.

117. Torres-Leguizamon M, Favaro J, Coello D, Reynaud EG, Néfau T, Duplessy C. Remote harm reduction services are key solutions to reduce the impact of COVID-19-like crises on people who use drugs: evidence from two independent structures in France and in the USA. *Harm Reduct J*. 2023;20(1):1.

118. Tsai YS, Kozman MS, Becker D, Lin JC, Xiang AH. Trends in adolescent depression screening outcomes over the COVID-19 pandemic at a large, integrated health care system in Southern California. *J Adolesc Health*. 2024;75(6):912-920.

119. Valicenti-McDermott M, O’Neil M, Morales-Lara A, Seijo R, Fried T, Shulman L. Remote learning experience for children with developmental disabilities during COVID-19 pandemic in an ethnically diverse community. *J Child Neurol*. 2022;37(1):50-55.

120. Watts R, Pattnaik J. Perspectives of parents and teachers on the impact of the COVID-19 pandemic on children’s Socio-emotional well-being. *Early Child Educ J*. 2022;51(8):1-12.

121. Weaver RG, Hunt ET, Armstrong B, et al. COVID-19 leads to accelerated increases in children’s BMI z-score gain: An interrupted time-series study. *Am J Prev Med*. 2021;61(4):e161-e169.

122. Weissman JD, Kramsky JB, Pinder N, Jay M, Taylor J. An examination of mental health rates in children during the first year of the COVID-19 pandemic: Findings from the National Health Interview Survey 2019-2020. *Child Psychiatry Hum Dev*. Published online September 18, 2024. doi:10.1007/s10578-024-01759-2

123. Weleff J, Anand A, Squeri M, Sieke R, Thompson NR, Barnett BS. An analysis of benzodiazepine prescribing to primary care patients in a large healthcare system from 2019-2020. *J Psychoactive Drugs*. 2024;56(2):245-256.

124. Whyte F, Chan WY, Silverstein DK, Conrad H, Lee B. Dental utilization in a pediatric emergency department and urgent care centers before, during, and after shutdown of a pediatric dental clinic during the COVID-19 pandemic, 2019-2021. *Public Health Rep*. 2023;138(3):493-499.

125. Wolf ER, Nguyen M, Sabo RT, et al. School closure and child maltreatment during the COVID-19 pandemic. *Child Maltreat*. 2024;29(3):500-507.

126. Yip SW, Jordan A, Kohler RJ, Holmes A, Bzdok D. Multivariate, transgenerational associations of the COVID-19 pandemic across minoritized and marginalized communities. *JAMA Psychiatry*. 2022;79(4):350-358.

127. Zablotsky B, Black LI, Terlizzi EP, Vahratian A, Blumberg SJ. Anxiety and depression symptoms among children before and during the COVID-19 pandemic. *Ann Epidemiol*. 2022;75:53-56.

128. Zacher NC, Pickett KL, Schmiege SJ, Olson CA, Bruckner AL, Kohn LL. Retrospective chart review of patient socioeconomic status and language preference associated with live video telehealth in a pediatric dermatology practice. *Pediatr Dermatol*. 2023;40(4):651-654.

129. Zeppieri G Jr, Hung CJ, Pazik M, Moser M, Farmer K, Pozzi F. The COVID-19 lockdown as a model of detraining in division 1 college softball players. *BMC Sports Sci Med Rehabil*. 2024;16(1):43.

130. Zhai M, Bono K, Zhang WW, et al. Drug and alcohol use in trauma patients before and during the COVID-19 pandemic. *J Surg Res*. 2023;283:999-1004.

131. Zhang X, Oluyomi A, Woodard L, et al. Individual-level determinants of lifestyle behavioral changes during COVID-19 lockdown in the United States: Results of an online survey. *Int J Environ Res Public Health*. 2021;18(8):4364.

132. Zima BT, Edgcomb JB, Rodean J, et al. Use of acute mental health care in U.s. children’s hospitals before and after statewide COVID-19 school closure orders. *Psychiatr Serv*. 2022;73(11):1202-1209.

**Supplementary Material D -** Among vulnerable groups, frequency of detrimental outcomes within studies examining the effect of U.S. COVID-19 lockdowns and/or school closures

|  | Racial/  ethnic minorities (n=29) | Low socioeconomic groups  (n=23) | At-risk youth (n=11) | Those with disabilities (n=11) | Elderly (n=9) | Non-English speaking/  immigrants (n=7) | Veterans (n=5) | Publicly insured/  uninsured (n=5) | Sexual minority group (n=4) | Total Detrimental |
| --- | --- | --- | --- | --- | --- | --- | --- | --- | --- | --- |
| Category of Outcome |  |  |  |  |  |  |  |  |  |  |
| Access to health services | 4 of 4 | 4 of 4 |  |  | 7 of 8 | 2 of 2 | 3 of 4 | 2 of 2 |  | 22 of 24 |
| Obesity | 10 of 10 | 3 of 3 | 1 of 1 |  |  | 1 of 1 |  | 3 of 3 |  | 18 of 18 |
| Mental health | 3 of 3 | 3 of 3 | 3 of 4 | 2 of 2 |  |  |  |  |  | 11 of 12 |
| Healthy behaviors | 0 of 1 | 4 of 4 |  | 2 of 2 |  |  |  |  | 3 of 3 | 9 of 10 |
| Alcohol/Drug/Substance Use | 1 of 1 |  | 4 of 6 |  |  | 1 of 1 |  |  | 0 of 1 | 6 of 9 |
| Child Developmental/Education |  | 3 of 4 |  | 3 of 3 |  | 2 of 2 |  |  |  | 8 of 9 |
| Economic/Financial stability | 3 of 3 | 3 of 3 |  |  |  |  |  |  |  | 6 of 6 |
| General health measures |  | 1 of 1 |  | 3 of 3 | 1 of 1 |  |  |  |  | 5 of 5 |
| Access to food | 3 of 3 |  |  |  |  | 1 of 1 |  |  |  | 4 of 4 |
| Disease-related |  |  |  | 1 of 1 |  |  | 0 of 1 |  |  | 1 of 2 |
| Employment | 2 of 2 |  |  |  |  |  |  |  |  | 2 of 2 |
| Suicide or Self-harm | 1 of 1 |  |  |  |  |  |  |  |  | 1 of 1 |
| Family well-being | 0 of 1 |  |  |  |  |  |  |  |  | 0 of 1 |
| Trauma/Injury |  | 1 of 1 |  |  |  |  |  |  |  | 1 of 1 |
| Total detrimental | 27 of 29 | 22 of 23 | 8 of 11 | 11 of 11 | 8 of 9 | 7 of 7 | 3 of 5 | 5 of 5 | 3 of 4 | 94 of 104 |

**Supplementary Material E.** Results from quasi-experimental studies (n=6) examining the effects of U.S. COVID-19 lockdowns and school-closures

| **Author (year)** | **Intervention** | **Study design** | **Outcome Category** | **Outcome (Type)** | **Direction** | **Effect** | **Additional context from manuscript** |
| --- | --- | --- | --- | --- | --- | --- | --- |
| Brodeur (2021) | Lockdowns | Difference-in-difference & Regression Discontinuity | Mental health | Google search for word “boredom” | Increased | Detrimental | “The evidence of a substantial increase in the search intensity on boredom, sadness, loneliness, and worry …suggests that people’s mental health has been adversely affected” |
|  |  |  | Mental health | Google search for word “loneliness” | Increased | Detrimental |  |
|  |  |  | Mental health | Google search for word “sadness” | Increased | Detrimental |  |
|  |  |  | Mental health | Google search for word “worry” | Increased | Detrimental |  |
|  |  |  | Mental health | Google search for word “boredom” | Increased | Detrimental |  |
|  |  |  | Mental health | Google search for word “stress” | Decreased | Beneficial |  |
|  |  |  | Suicide or Self-harm | Google search for word “suicide” | Decreased | Beneficial |  |
| Berger (2021) | Lockdowns | Panel event-study design & difference-in-differences | Healthy Behaviors | Google search for “condom” | Decreased | Unclear | The decrease in terms related to sexual behavior, contraceptive use, and pregnancy termination “may reflect a decrease in (unprotected) sexuality activity, perhaps particularly among those not experiencing lockdown with a coresident partner.” |
|  |  |  | Family Well-being | Google search for “emergency contraceptive pill” | Decreased | Unclear |  |
|  |  |  | Family Well-being | Google search for “pregnancy test” | Decreased | Unclear |  |
|  |  |  | Family Well-being | Google search for “abortion” | Decreased | Unclear |  |
|  |  |  | Family Well-being | Google search for “planning first child” | No change | Null |  |
|  |  |  | Family Well-being | Google search for “planning other children” | No change | Null |  |
| Ferwana (2024) | Both school closures and lockdowns | Difference in Differences | Mental health | Mental health facility use | Increase | Detrimental | “Results show that lockdown has significantly and causally increased the usage of mental health facilities…by 18% in regions with a lockdown compared to 1% decline in regions without a lockdown.”  “Diagnosis of panic disorders and reaction to severe stress significantly increased by the lockdown. Mental health was more sensitive to lockdowns than to the presence of the pandemic itself.” |
|  |  |  | Mental health | Mental health facility use among females compared to males | Increase | Detrimental |  |
|  |  |  | Mental health | Panic Disorder Diagnosis | Increase | Detrimental |  |
|  |  |  | Mental health | Severe Stress Diagnosis | Increase | Detrimental |  |
| Cafferty (2024) | Both school closures and lockdowns | Interrupted time series analysis | Suicide or Self-harm | Rate of positive suicide screens among youth aged 10-18 | Increased | Detrimental | There was a steady rise in the rate of positive suicide screenings each week during year one of the pandemic. School reopening and normalization of social routines preceded a decline in the rate of positive suicide screens in year 2 suggesting social connectedness and access to school-based resources may be a protective factor for youth suicide risk. |
| Rapoport (2021) | Both school closures and lockdowns | Seasonal Autoregressive Integrated Moving Average Models | Interpersonal violence/neglect/abuse | Reports of allegations of child maltreatment | Decreased | Detrimental | “Our findings demonstrate a marked decrease in child maltreatment reporting and provision of CPS inventions in NYC during the COVID-19 pandemic; if reflective of national trends, our study suggests that nationally, over a quarter million cases of child abuse or neglect have gone unreported.” |
|  |  |  | Interpersonal violence/neglect/abuse | Child protective service investigations | Decreased | Detrimental |  |
| Weaver (2021) | School closures | Interrupted time series analysis | Obesity | BMI z-score change among all children | Increased | Detrimental | “Before the COVID-19 pandemic, children’s yearly BMI z-score change was +0.03 (95%  CI= 0.10, 0.15). Change during the COVID-19 pandemic was +0.34 (95% CI=0.21, 0.47), an  acceleration in BMI z-score change of +0.31 (95% CI=0.19, 0.44).”  “Children’s zBMI-score was approximately 10 times greater during the COVID-19 pandemic than in the previous years."   - Yearly BMI z-score change accelerated in girls (+0.33 (95% CI=0.16, 0.50). - Yearly BMI z-score change accelerated in boys (+0.29, 95% CI=0.12, 0.46). - Yearly BMI z-score change accelerated in children who were Black (+0.41, 95% CI=0.21, 0.61). - Yearly BMI z-score change accelerated in children who were White (+0.22, 95% CI=0.06, 0.39). - Yearly BMI z-score change accelerated in children who were classified as normal obesity-related outcome weight (+0.58, 95% CI=0.40, 0.76). - Yearly BMI z-score change accelerated for lower elementary/primary (+0.23, 95% CI=0.08, 0.37) children. - Yearly BMI z-score change accelerated for upper elementary/primary (+0.42, 95% CI=0.42, 0.63) children. |
|  |  |  | Obesity | BMI z-score change among girls | Increased | Detrimental |  |
|  |  |  | Obesity | BMI z-score change among boys | Increased | Detrimental |  |
|  |  |  | Obesity | BMI z-score change among children who are Black | Increased | Detrimental |  |
|  |  |  | Obesity | BMI z-score change among children who are White | Increased | Detrimental |  |
|  |  |  | Obesity | BMI z-score change among children who were classified as having pre-pandemic normal weight. | Increased | Detrimental |  |
|  |  |  | Obesity | BMI z-score change among children in lower elementary grades | Increased | Detrimental |  |
|  |  |  | Obesity | BMI z-score change among children in upper elementary grades | Increased | Detrimental |  |
